# Supplementary material for: Cholinergic degeneration in prodromal and early Parkinson’s disease: a link to present and future disease states
Source: Brain. 2025 May 6;149(1):226–37. doi: 10.1093/brain/awaf168 (PMC12782177; doi:10.1093/brain/awaf168)
Supplement: awaf168_Supplementary_Data [file awaf168_supplementary_data.pdf]

Supplementary Information

Supplementary Table 1. Contribution of each cohort to the study groups' participants

Cohort	PD (N = 393)	iRBD (N = 128)	HC (N = 186)
ODC	105	80	59
PPMI	288	48	127

Supplementary Table 2. demographic, cognitive, and clinical characteristics of study participants (mean±SD and proportions are presented for continuous and categorical variables, respectively).

Characteristic	PD (N = 393)	PD + pRBD (N = 104)	PD - pRBD (N = 286)	iRBD (N = 128)	HC (N = 186)
Age (years)	63.5 (9.54)	64.7 (9.06)	63.0 (9.63)	67.2 (7.23)	62.6 (10.91)
Sex					
Female	149 (38%)	25 (24%)	121 (42%)	13 (10%)	68 (37%)
Male	244 (62%)	79 (76%)	165 (58%)	115 (90%)	118 (63%)
Education (years)	15.7 (3.12)	15.6 (3.03)	15.8 (3.16)	14.4 (3.40)	16.0 (3.09)

Characteristic	PD (N = 393)	PD + pRBD (N = 104)	PD - pRBD (N = 286)	iRBD (N = 128)	HC (N = 186)
MoCA score	27.3 (2.10)	27.1 (2.23)	27.3 (2.06)	26.3 (2.30)	27.9 (2.00)
Higher order cognition (z-score)	-0.01 (0.64) (N = 282)	-0.19 (0.66) (N = 209)	0.06 (0.63) (N = 72)	0.00 (0.68) (N = 48)	-
UPDRS-1 total score	6.4 (4.30)	6.9 (4.37)	6.2 (4.28)	8.5 (5.13)	
UPDRS-3 total score	22.8 (9.68)	24.5 (11.18)	22.3 (9.04)	4.1 (3.37)	-
Disease duration (months)	9.1 (8.96)	9.1 (8.93)	9.0 (8.88)	27.3 (44.37)	-
TIV (ml)	1,531.2 (152.86)	1,550.7 (127.59)	1,525.7 (160.43)	1,541.1 (125.34)	1,495.9 (146.81)
IQR	80.7 (3.66)	80.6 (3.82)	80.7 (3.61)	81.6 (3.95)	80.3 (3.74)

PD + pRBD = PD with possible RBD; PD - pRBD = PD without possible RBD

Supplementary Table 3. Between-group differences in individual cognitive tests.

Test	Between-group comparison (iRBD vs. PD)
Letter-Number Sequencing test	β=-0.06 [95% CI -0.39, 0.27], *p*=0.71
Benton Judgment of Line Orientation test	β=0.06 [95% CI -0.25, 0.38], *p*=0.69
Semantic Verbal Fluency test	β=0.29 [95% CI -0.03, 0.62], *p*=0.08
Symbol Digit Modalities Test	β=0.30 [95% CI -0.03, 0.62], *p*=0.08
Hopkins Verbal Learning Test delayed	β=-0.03 [95% CI -0.37, 0.31], *p*=0.86
Hopkins Verbal Learning Test immediate	β=-0.08 [95% CI -0.40, 0.25], *p*=0.65

Supplementary Table 4. Associations between NbM volume and individual higher-order cognitive tests.

Test	iRBD	PD
Letter-Number Sequencing test	β=0.08 [95% CI -0.28, 0.44], *p*=0.67	β=0.08 [95% CI -0.09, 0.24], *p*=0.36
Benton Judgment of Line Orientation test	β=0.15 [95% CI -0.26, 0.55], *p*=0.50	**β=0.27 [95% CI 0.11, 0.44], *p*=0.002**
Semantic Verbal Fluency test	β=0.35 [95% CI -0.03, 0.72], *p*=0.10	β=-0.02 [95% CI -0.17, 0.14], *p*=0.85
Symbol Digit Modalities Test	β=0.01 [95% CI -0.35, 0.37], *p*=0.95	**β=0.20 [95% CI 0.05, 0.35], *p*=0.01**
Hopkins Verbal Learning Test delayed	β=0.03 [95% CI -0.32, 0.38], *p*=0.86	β=-0.004 [95% CI -0.16, 0.15], *p*=0.96
Hopkins Verbal Learning Test immediate	β=0.07 [95% CI -0.28, 0.42], *p*=0.70	β=0.06 [95% CI -0.09, 0.20], *p*=0.45

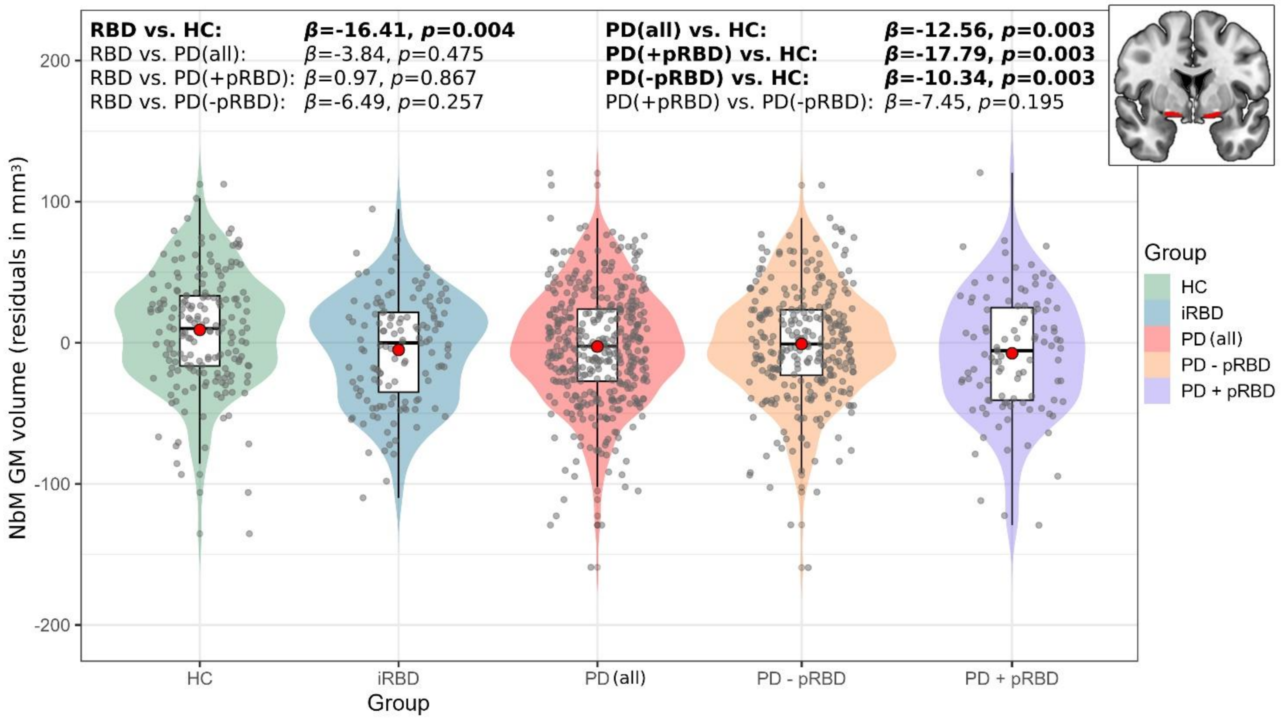

Supplementary Figure 1. Between-group differences in grey matter volume of the NbM. When dividing the PD group based on the RBDSQ score, both PD + pRBD (β=-17.79 [95% CI -27.92, -7.66], *pFDR*=0.003) and PD – pRBD (β=-10.34 [95% CI -18.16, -2.52], *pFDR*=0.003) demonstrated lower NbM volume compared to controls, with no difference between the iRBD and PD groups (*pFDR*>0.19 for all pairwise comparisons). Red circles within boxplots represent group mean. Upper right corner: bilateral NbM mask in red. Group-differences with *p*<0.05 are highlighted with bold. P-values are FDR-corrected. PD + pRBD = PD with possible RBD; PD - pRBD = PD without possible RBD
